# Ventriculitis due to *Staphylococcus lugdunensis*: two case reports

**DOI:** 10.1186/1752-1947-2-267

**Published:** 2008-08-11

**Authors:** Teresa Spanu, Donato Rigante, Gianpiero Tamburrini, Barbara Fiori, Tiziana D'Inzeo, Brunella Posteraro, Domenico Policicchio, Maurizio Sanguinetti, Giovanni Fadda

**Affiliations:** 1 Institute of Microbiology, Catholic University of the Sacred Heart, Largo A. Gemelli 8, 00168, Rome, Italy; 2 Department of Pediatric Sciences, Catholic University of the Sacred Heart, Rome, Italy; 3 Department of Pediatric Neurosurgery, Catholic University of the Sacred Heart, Rome, Italy

## Abstract

**Introduction:**

*Staphylococcus lugdunensis *is an unusually virulent coagulase-negative staphylococcus that has rarely been implicated in central nervous system infections.

**Case presentation:**

Two children hospitalized in the Neurosurgery Unit developed ventriculitis caused by methicillin-resistant *Staphylococcus lugdunensis *following placement of external ventriculostomy drains. The causative organisms were identified by molecular studies. The patients recovered without significant sequelae after high doses of intrathecal vancomycin.

**Conclusion:**

Distinguishing *Staphylococcus lugdunensis *from other coagulase-negative staphylococcus species is crucial because it carries a substantial risk for severe central nervous system infections displayed by patients with implanted cerebrospinal fluid devices. Clinicians should not underestimate the importance of the isolation of this species from cerebrospinal fluid specimens.

## Introduction

First described by Freney *et al. *in 1988 [1], *Staphylococcus lugdunensis *is an unusually virulent coagulase-negative staphylococcus (CoNS) known primarily as a cause of endocarditis, especially in immunocompromised patients [2]. It has also been associated with septic arthritis, osteomyelitis, peritonitis, brain abscesses, and infections of the skin and soft tissues, urinary tract, and prosthetic medical devices [3]. Its remarkable virulence has been attributed to the production of extracellular slime, which facilitates colonization and interferes with phagocytosis-associated activities of neutrophils [4]. Some strains also produce a synergistic hemolysin that resembles the δ-hemolysin of *S. aureus*, consisting of three very similar 43-residue peptides closely related to the gonococcal-growth-inhibitor bacteriocin secreted by *S. haemolyticus*. Nucleic acid sequences related to the accessory gene regulator (the major determinant of virulence in *S. aureus*) have also been demonstrated in *S. lugdunensis *[5].

A MEDLINE search conducted with the keywords "*S. lugdunensis*" *AND *"cerebrospinal fluid (CSF)-shunt infection" *OR *"central nervous system (CNS) infection" yielded only four cases, which are summarized in Table 1. Cases #1 through #3 were *S. lugdunensis *ventriculo-peritoneal shunt (VPS) infections [6,7], and case #4 was a *S. lugdunensis *meningitis unrelated to implanted CSF drainage devices [8]. The other two cases of *S. lugdunensis *infections reported in Table 1 were recently diagnosed in the Pediatric Neurosurgery Unit of our Medical Center.

**Table 1:** Clinical data and outcome of ventriculitis or meningitis caused by *Staphylococcus lugdunensis*

Case # Gender – Age	Underlying disease	CSF shunt^a^	Time to infection^b^	Signs and symptoms	Antimicrobial therapy	Outcome	Reference (year)
Case #1 M – 74 years	Ventriculomegaly	VPS	14 days	Fever, abdominal pain, sweating	Vancomycin^c ^+ rifampicin + cefuroxime, then vancomycin^c ^+ rifampicin, then ciprofloxacin + rifampicin	Recovered	Sandoe (2001)
Case #2 F – 10 months	Obstructive hydrocephalus	VPS	3 days	Fever, irritability, decreased activity	Oxacillin	Recovered	Elliott (2001)
Case #3 F – 16 years	Aqueduct stenosis	VPS	2 years	Fever, lethargy, abdominal complaint	Vancomycin^c^ then oxacillin	Recovered	Elliott (2001)
Case #4 M – 12 years	Obstructive hydrocephalus	ND^d^	-	Fever, headache, vomiting, lethargy	Vancomycin^c ^+ cefotaxime, then oxacillin + rifampicin	Recovered	Kaabia (2002)
Case #5 M – 7 years	Tumor within the posterior cranial fossa	EVD	20 days	Fever, headache, vomiting, lethargy	Vancomycin^e^	Recovered	This study
Case #6 M – 2 months	Malformation of Galen's vein	EVD	19 days	Fever, seizures, impaired consciousness	Vancomycin^e^	Recovered	This study

^a^CSF shunt, cerebrospinal fluid shunt included VPS or EVD.

^b^From shunt placement to infection onset.

^c^Administered intravenously.

^d^ND = not described.

^e^Administered intrathecally.

This report analyzes the management of these patients in light of the few previously reported cases of *S. lugdunensis *CNS infections and summarizes the molecular characteristics of the isolates recovered from CSF and ventricular drain cultures.

## Case presentation

### Patient 1

A 7-year-old boy was hospitalized in the Pediatric Neurosurgery Unit for headache, vomiting, and right ocular dysmetria. Magnetic resonance imaging (MRI) revealed obstructive hydrocephalus caused by a posterior fossa tumor. The child was taken to the operating room for placement of an external ventriculostomy drain (EVD). CSF cultures yielded no growth. There was no improvement and, on the 10^th ^day of hospitalization, he had a second operation for partial removal of the tumor (a medulloblastoma). A new EVD was inserted; cultures of the original EVD and CSF yielded no growth. Seven days later, the child developed severe headache, fever (39.5°C), vomiting, lethargy, and signs of EVD malfunction. Shunt puncture yielded cloudy CSF containing 900 leukocytes/mm^3 ^(80% polymorphonuclear cells); 100 mg protein/dl); and 21 mg glucose/dl (blood glucose: 87 mg/dl). Cytocentrifuge Gram staining revealed Gram-positive cocci that were later identified by biochemical and molecular methods as *Staphylococcus lugdunensis*.

Meanwhile, a presumptive diagnosis of ventriculitis was made [9], a new EVD was inserted, and intrathecal (IT) vancomycin (40 mg/day) was started. Cultures of the CSF and the ventricular tip of the second EVD grew methicillin-resistant *S. lugdunensis*. Three blood cultures yielded no growth. Swabs of both inguinal folds and the surgical incision were cultured, but none yielded *S. lugdunensis*. Defervescence occurred after 2 days of IT vancomycin. After 14 days of vancomycin, the composition of the CSF was normal, and cultures of CSF and EVD were negative. The boy was discharged after 62 days of hospitalization, and no sign of CNS infection was noted at the 6-month follow-up visit.

### Patient 2

A 2-month-old male was admitted to the Pediatric Neurosurgery Unit for rapid head growth with a tense anterior fontanel. He was placed in the same room where Patient 1 was still being cared for. Triventricular hydrocephalus was evident on cranial ultrasonography, and MRI with angiographic sequences disclosed a malformation involving Galen's vein that caused arteriovenous shunting and obstruction of CSF flow through the aqueduct. The malformation was treated successfully with intravascular embolization, but 1 week later, the infant developed seizures. Cerebral angiography confirmed occlusion of the aneurysmal malformation, but computed tomography revealed an intraventricular/subarachnoid hemorrhage. An EVD was placed for continuous intracranial pressure monitoring and collection of CSF specimens. All CSF cultures were negative, including the one obtained during EVD placement. Three days later, the child was febrile (39.2°C) and drowsy. A cloudy CSF specimen collected from the EVD contained 129 mg/dl protein, 20 mg/dl glucose (blood glucose: 113 mg/dl), 800 leukocytes/mm^3 ^(60% were polymorphonuclear cells), and 5 to 10 Gram-positive cocci per microscopic field that were identified in biochemical and molecular assays as *S. lugdunensis*.

The clinical picture was compatible with ventriculitis [9], and IT vancomycin (40 mg/dose/day) was started immediately after EVD replacement and continued for 14 days. Cultures of the CSF and the tip of the original EVD grew methicillin-resistant *S. lugdunensis*. Three blood cultures were negative. Skin cultures (inguinal folds, surgical incision site) were all negative for *S. lugdunensis*. The infant's condition rapidly improved after vancomycin was started, and cultures of CSF collected 15 days later and of the second EVD showed no growth. The infant was discharged after 40 days of hospitalization. Six months later, he was well with no evidence of infection and no acute neurological signs.

### Microbiological diagnosis

Our routine protocol for suspected CSF shunt infections includes Gram staining of cytocentrifuged CSF, aerobic culture at 35°C on MacConkey agar, microaerobic culture (35°C in room air with 5% CO_2_) on Columbia and chocolate agars, anaerobic culture (35°C) on Schaedler agar (all from bioMérieux, Marcy-L'Etoile, France), and 72 hours of aerobic and anaerobic cultures on brain-heart infusion broth supplemented with 5% NaCl.

In these two patients, cultures of CSF and EVD tips on Columbia agar produced yellowish beta-hemolytic colonies (diameter: 0.8–2.5 mm) of Gram-positive cocci. Tube coagulase tests with rabbit plasma (Becton Dickinson Microbiology Systems, Sparks, MD) were negative. The isolates were positive for catalase, clumping factor, pyrrolidonyl arylamidase, and ornithine decarboxylase. They were identified as *S. lugdunensis *by the API ID32 STAPH (bioMérieux) and the VITEK 2 (bioMérieux, Inc, Hazelwood, MO) systems.

Bacterial DNA was extracted from CSF specimens and culture isolates with the QIAmp DNA Mini kit (QIAGEN, Hilden, Germany). Species-level identification was confirmed by sequencing of the 16S rRNA gene (using the RIDOM entries) and the RNA polymerase B gene [10]. Sequence analysis of the 16S rRNA gene revealed 100% homology with the prototype strain sequence of *S. lugdunensis *ATCC 43809 (Z26899). A partial sequence of the *rpoB *gene of each isolate revealed 99.5% homology with the prototype strain sequence of *S. lugdunensis *ATCC 43809 (EF173667).

*A*ntimicrobial susceptibility testing with the E-test (AB Biodisk, Solna, Sweden) yielded the following MICs (μg/ml): oxacillin, 256; vancomycin, 0.5; erythromycin, 0.03; ciprofloxacin, 0.03; clindamycin, 0.03; rifampicin, 4.0; quinupristin-dalfopristin, 0.5; linezolid, 1.0. Resistance was defined by Clinical Laboratory Standards Institute breakpoints [11]. The *mecA *gene (reflecting methicillin resistance) was detected by PCR, as described by Geha and colleagues [12]. Pulsed-field gel electrophoresis (PFGE) analysis of *Sma*I- and *Apa*I-digested chromosomal DNA [13] revealed identical profiles for all isolates recovered from Patients 1 and 2 (data not shown).

## Discussion

To date, there have been no reports of *S. lugdunensis *ventriculitis associated with EVDs, which are widely used in neurosurgery for continuous intracranial pressure monitoring, injection of therapeutic agents, and temporary external drainage of CSF [9]. EVD-associated CSF infections can be classified as ventriculostomy-related infections or ventriculitis [9]. The former are generally associated with few clinical symptoms. The CSF is characterized by culture- or Gram-stain-positivity with progressive decrease in glucose and progressive increase in proteins. Progression to ventriculitis is heralded by high-grade fever and signs of meningitis (for example, nuchal rigidity, photophobia, deteriorating mental status, seizures, moribund appearance). The latter pattern was seen in both of our patients. Fever and meningeal signs were also reported in cases #2, 3 and 4 (Table 1), whereas the VPS infection in case #1 was associated with severe non-neurological symptoms (intra-abdominal sepsis with purulent peritonitis) [7].

Our patients and one of those reported by Elliot and colleagues [6] had hospital-acquired infections, which developed 3 to 7 days after shunt insertion. The other infections shown in Table 1 were evident at hospital admission and would thus seem to be community-acquired (although in one case [7] the admission occurred 14 days after a previous hospitalization during which a CSF shunt had been inserted).

EVD use in critically ill neurosurgical patients is on the rise and reported rates of infection associated with these devices vary widely (from 0 to 45%). The prevailing opinion is that the infecting agent is usually introduced during EVD placement [9], which is consistent with the fact that most EVD-associated infections are caused by skin flora, particularly *Staphylococcus epidermidis*.

Like other CoNS species, *S. lugdunensis *is considered part of the resident flora of the human skin and mucous membranes, although the preferred carriage site seems to be the perineum [14]. In both of our patients, infection onset occurred a few days after surgery, the patients were being cared for in the same hospital room, and all isolates displayed the same pulsotype. These findings are suggestive of a common source, which unfortunately has not been identified.

We cannot exclude the possibility that the infections were transmitted during manipulation of the catheters. Healthcare-provider hand cultures (data not shown) and patient skin cultures yielded no growth of *S. lugdunensis*. However, the staff surveillance cultures were collected after the second child had been infected, not during the time of EVD insertion, so the infection reservoir might have been missed. Environmental cultures and inguinal cultures of providers were not performed.

Hellbacher and colleagues [13] recently suggested that PFGE is unsuitable for analyzing outbreak situations involving *S. lugdunensis*. The homogeneity they observed among 39 isolates collected over 4 years suggests that *S. lugdunensis *is either a highly conserved species or that specific clones are more likely to cause invasive infections. Other investigators [14], however, have found that PFGE with *Sma*I macrorestriction analysis is inappropriate for epidemiological investigations of *S. lugdunensis *infections only when the strains are β-lactamase-producers, since these isolates usually display a high level of genetic homogeneity. Identification of adequate typing tools for this bacterial population will probably require multimodal molecular characterization of a larger collection of *S. lugdunensis *strains.

The standard approach to CSF shunt infections caused by a methicillin-resistant *Staphylococcus *spp. includes shunt removal and intravenous vancomycin [9]. The previously reported CSF infections caused by *S. lugdunensis *were treated with intravenous vancomycin and/or oxacillin, alone or with rifampicin, and bacteriological and clinical cures were documented in all four [6-8]. CSF shunt infections caused by methicillin-resistant staphylococci are common in our hospital, so when Gram-stain data indicated the possibility of staphylococcal ventriculitis, our patients were both treated empirically with vancomycin. Later, the isolates' methicillin resistance was confirmed by susceptibility testing and PCR analysis. Our patients were treated with intrathecal vancomycin (40 mg/day), and bacteriological and clinical cures were achieved in both cases. Since then, guidelines have been published in which considerably lower doses are recommended for children (for example < 5 mg/24 hours) [15]. Although no adverse effects were experienced by our patients, we have modified our protocol, and staphylococcal CSF shunt infections are now treated with IT vancomycin at the dosage indicated above and with drug levels closely monitored.

The frequency of *S. lugdunensis *infections may well be underestimated. The species is likely to escape detection by screening tests or certain automated microbiological systems. Its ability to produce colony variations is well known, described in three of the 11 strains included in the initial description of *S. lugdunensis *in 1988 [1]. Awareness of this risk has led to the development of genetic tools for identifying this species [7]. No colony variation was observed in either of our patients and all isolates consistently exhibited the normal *S. lugdunensis *phenotype in blood agar cultures.

## Conclusion

Our experience confirms that, unlike other CoNS which usually display low virulence, *S. lugdunensis *can cause severe CNS infections in patients with implanted CSF drainage devices. Accurate species-level identification of isolates causing staphylococcal CSF shunt infections is clearly essential for their successful treatment, but it is also fundamental for epidemiological surveillance and for improving our understanding of the pathophysiological factors affecting the clinical outcome of these infections. With the increasing use of invasive medical devices for management of neurosurgical patients, CSF shunt infections are likely to become more common. Failure to identify their causative agents can be particularly disastrous when the infection is due to *S. lugdunensis*.

### Nucleotide sequence accession numbers

The sequences of the isolates evaluated in this study have been deposited in the GenBank database under accession nos. FM177467 and FM177468, respectively, for the *rpoB *gene and under accession nos. FM177469 and FM177470, respectively, for the 16 rRNA gene.

## Abbreviations

CNS: Central nervous system; CoNS: Coagulase-negative staphylococcus; CSF: Cerebrospinal fluid; EVD: External ventriculostomy drain; IT: Intrathecal; VPS: ventriculo-peritoneal shunt; MRI: Magnetic resonance imaging; PFGE: Pulsed-field gel electrophoresis.

## Competing interests

The authors declare that they have no competing interests.

## Authors' contributions

TS and DR participated in the conception and design of the study, acquisition of data, analysis and interpretation of data, drafting of the manuscript and its critical revision. GT and DP managed both children as neurosurgeons. BF, TDI, BP and MS carried out the laboratory studies of patients. GF revised the manuscript. All authors read and approved the final version of the manuscript.

## Consent

Written consent was obtained from each patient's parents for the publication of this report. A copy of the written consent is available for review by the Editor-in-Chief of this journal.
